# The outcome of selective delayed sentinel lymph node biopsy following upfront omission of axillary staging in low-risk invasive breast cancers: a retrospective hypothetical simulated analysis

**DOI:** 10.1007/s12672-025-03344-y

**Published:** 2025-08-12

**Authors:** Albin Bengtsson, Karolina Larsson, Kian Chin

**Affiliations:** 1 Department of Surgery, Kungälv Hospital, Gothenburg, Sweden; 2 Department of Oncology, Sahlgrenska University Hospital, Gothenburg, Sweden; 3 Department of Surgery, Sahlgrenska University Hospital, Gothenburg, Sweden; 4 Institute of Clinical Sciences, Sahlgrenska Academy at Gothenburg University, Gothenburg, Sweden

**Keywords:** Breast cancer, Luminal A-like cancer, Sentinel lymph node biopsy, Axillary lymph node dissection, Metastases, Radiotherapy, Chemotherapy

## Abstract

**Background:**

Sentinel lymph node biopsy (SLNB) is performed to guide recommendations on adjuvant treatments for invasive breast cancer. However, studies have shown oncological safety without SLNB in low-risk patients. We aimed to determine the clinical benefits of delaying SLNB (d-SLNB), if upfront axillary staging was omitted in patients with low-risk invasive breast cancers.

**Methods and materials:**

A retrospective hypothetical simulated analysis. Patients who had breast surgery and SLNB between 2019 and 2021 were included. Patients with low-risk invasive cancers were identified based on preoperative histopathology (≥ 65 years, Luminal A-like, T1, cN0, Grade 1–2). Outcome analyses were based on the Actual clinical management compared to two different hypothetical Scenarios: (A) upfront SLNB omission only, and (B) upfront SLNB omission with d-SLNB. Primary endpoints were proportion of patients suitable for SLNB omission, outcome of d-SLNB and changes in adjuvant treatments. Secondary endpoint was surgical costs.

**Result:**

Of 712 patients, 205 (30%) had low-risk invasive cancers and eligible for SLNB omission. In Scenario A, 25 (12%) patients with SLN metastases would have understaged. If Scenario B was applied, the false negative rate of axillary staging would reduce from 25 (12%) to 12 (6%) patients, *p* < 0.001. On average, adjuvant treatments were given to 73% (Actual clinical setting) vs. 27% (Scenario A) vs. 55% (Scenario B), *p* < 0.001. Based on 100 patients, d-SLNB was associated with an incremental cost of 55,000 EUR per 100 patients.

**Conclusion:**

Although upfront SLNB omission was associated with missed SLN metastases, majority of low-risk invasive cancers were SLN negative. Delayed-SLNB could provide additional useful information to guide adjuvant treatments.

## Introduction

At the time of diagnosis of breast cancer, almost one third of all patients have axillary lymph-nodes metastases [1]. Despite routine axillary staging, it has been shown to convey no significant survival advantage in certain groups of patients with low-risk breast cancers. For example, the 10-year breast cancer-related survival in Sweden for low-risk luminal A-like cancers, can be favorable up to 95% [2]. More recent results from the SENOMAC trial confirmed no differences in 5-years recurrence free survival in patients with SLN metastases randomized to axillary lymph node dissection (ALND) or not it [3]. Earlier studies of patients with clinically node-negative (cN0) breast cancer like the NSABP-B32 trial, have also shown no differences in overall survival, disease-free survival or regional disease control when comparing ALND to SLNB [4]. Results from recent SOUND and INSEMA trials, showed that SLNB omission in low-risk patients was associated with non-inferior survival outcome [5, 6]. In 2021, in line with the campaign of Choosing Wisely to minimize non-beneficial use of resources in healthcare [7], the American Society of Clinical Oncology recommended that SLNB is not required for patients older than 70 years with small (T1) and cN0 invasive breast cancer that is estrogen receptor positive (ER-positive) and HER2-negative [8]. Therefore, the clinical value of SLNB in a subgroup of patients with low-risk invasive breast cancers is increasingly being challenged.

Currently, axillary lymph nodal status remains a much relied-upon prognostic factor, despite planning for adjuvant systemic treatment has become increasingly individualized based on tumor characteristics and biological subtypes as well as on gene expression analysis [9, 10]. For example, in Sweden within the context of breast conservation surgery, patients above 50 years of age with node negative disease will receive only five days of breast radiotherapy (RT) but in the presence of node positive disease, both breast and regional lymph nodes RT will be given over three weeks [11, 12]. Therefore, there can be persistent concerns amongst some clinicians regarding the missed SLN metastases if SLNB is omitted and the subsequent increased risk of regional recurrences [13]. On the other hand, there is currently the T-Rex study looking to avoid axillary RT with SLN metastases in order to avoid postoperative regional RT arm morbidities, pneumonitis and cardiac complications. The result of this study is currently not yet available [14]. The concept of selective delayed-SLNB (d-SLNB) after upfront omission of SLNB in low-risk patients with invasive ductal breast carcinoma can potentially be beneficial via ascertaining more information from the axilla to facilitate decision-making on adjuvant treatment. Therefore, in the context of upfront SLNB omission, we aimed to determine if selective d-SLNB could provide additional clinically useful information to guide adjuvant treatments in patients with initially low-risk invasive ductal cancers, that became no longer low-risk based on final histopathology on surgical specimens. The study was conducted in a retrospective hypothetically simulated clinical setting.

## Methods and materials

### Patients and clinical data

All patients who underwent surgery for invasive breast cancer at Sahlgrenska University Hospital between 2019 and 2021 were extracted from the hospital database system. The inclusion criteria for low-risk invasive cancers were ≥ 65 years at diagnosis, tumor size ≤ 20 mm (T1), estrogen receptor expression ≥ 10% (ER-positive according to National Swedish Treatment Guidelines), a pragmatic cut-off Ki67 index of ≤ 20%, Nottingham Histological Grade 1 to 2 and Human Epidermal Growth Factor-2 (HER2) negative (Hercept test 0 to 1+, 2 + with negative silver in-situ hybridization test and HER2 underdetermined) and clinically node negative (cN0) status based on ultrasound and clinical palpation. Tumor biology criteria for patient inclusion was based on preoperative histopathology from core biopsy. Preoperative tumor size was based on the largest measurement according to either mammography or ultrasound. Patients with suspicious axillary lymph nodes who underwent fine needle aspiration or core biopsy with benign results were considered cN0 and therefore included.

Exclusion criteria were patients who underwent surgery due to recurrent breast cancer, male gender, multifocal tumors, absence of preoperative core biopsy, presence of distant metastatic disease at diagnosis, Grade 3 or DCIS only. Details on postoperative tumor multifocality, which can be a prognostic marker [15], was not routinely available in all histopathology reports and was therefore not used as an exclusion criterion. Postoperative tumor size was based on the final histopathology report on the largest diameter of the invasive component. Pre- and postoperative tumor characteristics were presented in Table 1.

**Table 1:** Overall patient and tumor characteristics in a cohort with low-risk breast cancer. Preoperative and postoperative tumor biology based on core biopsy and resected specimens respectively were also shown

Whole group	*N*	
Number of patients	205	
Age, mean (range), years	72 (65–89)	
Types of surgery, No. (%)		
Breast conservation	178 (87)	
Mastectomy	27 (13)	
Median SLN removed, median (IQR)	2 (1–9)	
Patients with metastatic SLNs, No. (%)	25 (12)	

Comparison of tumor biology	Preoperative histopathology	Postoperative histopathology	p-value
Tumor size, mean (range), mm	11 (4–20)	14 (1–47)	< 0.001^a^
Hormone receptor status, No. (%)			
Positive	205 (100)	205 (100)	1.00
Negative	0 (0)	0 (0)	
Ki-67 (cut-off ≤ 20%), No. (%)			
Low	205 (100)	169 (82)	0.64
High	0 (0)	36 (18)	
Nuclear Grade, No. (%)			
1	69 (34)	59 (29)	< 0.001^b^
2	136 (66)	141 (69)	
3	0 (0)	5 (2)	
HER-2 status, No. (%)			
Positive	0 (0)	9 (5)	< 0.001^b^
Negative	152 (75)	196 (95)	
Undetermined	53 (25)	0 (0)	
Intrinsic subtypes, No. (%)			
Luminal A	205 (100)	164 (80)	< 0.001^b^
Luminal B	0 (0)	32 (16)	
HER-2 positive Luminal	0 (0)	9 (4)	

^a^Independent T-test,

^b^Fischer exact test. *P* < 0.05 was regarded as statistically significant

*ER* estrogen receptor, *HER-2* human epidermal growth factor receptor 2, *PR* progesterone receptor and *SLN* sentinel lymph node

### Study design and endpoints

This was a retrospective cohort study to determine if d-SLNB could be associated with clinical benefits by hypothetically omitting upfront SLNB in two simulated settings. The comparator would be outcome of SLNB in the Actual clinical setting. Two hypothetical scenarios were simulated: (A) upfront SLNB omission only, and (B) upfront SLNB omission with d-SLNB (Fig. 2). All patients identified with low-risk invasive cancers based on preoperative histopathology were separately assigned to both hypothetical scenarios. As there would be no SLNB performed in Scenario A, SLNs were presumed free from metastatic disease in all patients. The primary endpoints were: (1) proportion of patients who would be suitable for SLNB omission, (2) changes in axillary nodal status with hypothetical simulations compared to the Actual setting, and (3) changes in adjuvant treatments according to the Swedish National Guidelines [11] (see below) with hypothetical simulations compared to the Actual setting. The secondary endpoint was surgical costs. All endpoints were compared among the Actual clinical setting and the hypothetically simulated Scenarios A and B.

### Comparison of adjuvant treatments between actual clinical setting, scenario A and scenario B

Proportions of patients who received various categories of adjuvant treatments in the Actual setting were compared to those in Scenario A and B. The following adjuvant treatment categories were included for comparisons with the Swedish National Guidelines (version 2020) used as reference summarized in parentheses [11]: (1) Completion ALND, (patients with SLN metastases to undergo ALND), (2) endocrine therapy, patients with hormone receptor positive cancer to receive endocrine therapy: node negative patients (N0) treatment for 5 years and node positive (N+) treated with prolonged therapy), (3) Bisphosphonate therapy, patients with N + disease recommended bisphosphonate therapy, (4) breast and chest wall radiotherapy, N0 and N + after breast conserving surgery (BCS) whole breast radiotherapy without boost. N0/T1-T2 after mastectomy with radical margins: no chest wall RT. N + after mastectomy: chest wall RT, (5) axillary RT, N0 after BCS or mastectomy: no axillary RT. N + after BCS or mastectomy: axillary RT, and (6) chemotherapy, assume good performance status with low co-morbidities. N0 disease generally not recommended chemotherapy whereas it is considered if N+. Subtypes like luminal B, luminal A (1–3 N+) and triple negative breast cancers. Human epidermal growth factor receptor type 2 (HER2) positive (N0 or N+) were considered for chemotherapy. During the study period, usage gene profiling tests were not part of the national guidelines and not available.

Patients without SLN metastases were not included in analyses for changes in adjuvant treatments as the comparative outcome would not have been different due to unchanged nodal status, namely pre- and postoperative nodal status were the same. Although there were changes in subtypes based on final histopathology that may have impacted on adjuvant treatments, these patients were not included in the simulated analyses as the study aim was based on nodal status.

### Selection for d-SLNB

In Scenario B, after omitting upfront SLNB, patients were further assessed for low-risk invasive cancer status based on the final postoperative histopathology from surgery. If the tumor biology no longer fulfilled the above-mentioned criteria for low-risk cancer (i.e. non-low-risk), then a retrospective simulated d-SLNB was applied (Fig. 2).

### Cost calculations

To examine the difference in costs of surgery among Actual clinical setting and the two simulated Scenarios, relevant healthcare cost tariffs, used during the study period, were retrieved from the hospital’s financial database. The costs for lymphatic tracer, operation room, anesthesia, postoperative care, medications, histopathology assessments and radiology were included in the calculations based on BCS or mastectomy only, BCS or mastectomy plus SLNB, and SLNB alone. A subsequent d-SLNB would have a standalone unit operation cost.

### Statistical methods

Descriptive methods were used for presenting basic data on patient and tumor characteristics. Continuous data was compared using independent t-test. Categorical data was compared by using Chi-square and Fisher exact tests. Findings with a p-value of less than 0.05 was regarded as statistically significant. Confidence intervals of 95% were used. The statistical software IBM SPSS (statistical package for social science) version 29.0.2.0 (20) was used for all analyses.

## Result

### Patient and tumor characteristics

**Fig. 1:**
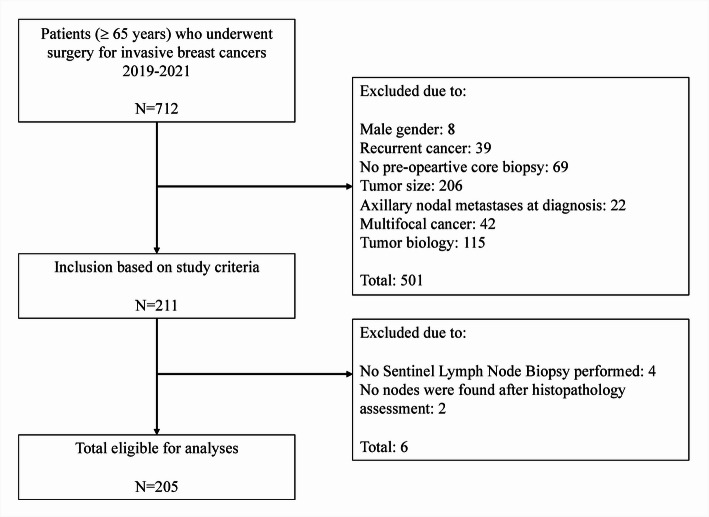
A flow diagram showing process of patient selection among all operated for invasive breast cancer between 2019–2021

A total of 712 patients, aged 65 years or older, underwent breast surgery for invasive breast cancer with SLNB between 2019 and 2021. Of these, 211 patients met the inclusion criteria as low-risk cancers for hypothetical omission of SLNB and 501 did not (Fig. 1). Six patients were further excluded due to no available information on SLN, as four never underwent SLNB and two underwent SLNB, but no nodes were found in the tissue specimens. Therefore, 205 of 712 (30%) patients were included for final analyses. Mean age was 72 years (65–89) and the majority underwent BCS (87%). All patients included in the final analysis underwent SLNB with a median of two lymph nodes removed. Comparing pre- and postoperative tumor characteristics, the tumors were found to be statistically significantly larger when measured on final histopathology in the surgical specimen (mean 14 mm) compared to imaging before surgery (mean 11 mm), *p* < 0.001. Thirty-six tumors (18%) were considered Ki67-high in the postoperative setting (*p* = 0.64). In total, five of 69 (15%) Grade 1 tumors were upgraded to Grade 3. Based on final histopathology of all these patients with initial low-risk Luminal A-like cancers, 32 (16%) were unexpectedly upstaged to Luminal B-like and nine (4%) were unexpectedly upstaged to HER2-positive tumors on final histopathology (*p* < 0.001) (Table 1). Patients with SLN metastases underwent more often mastectomy compared to those without SLN metastases: seven of 25 (28%) vs. 20 of 180 (11%), *p* = 0.06 respectively. These patients also had larger tumors: 22 mm vs. 14 mm, *p* < 0.001, more tumors with high Ki67 index: six of 25 (24%) vs. 30 of 180 (17%), *p* = 0.26, and higher proportions of Grade 2 and 3 tumors than those without SLN metastases: Grade 2: 22 of 25 (88%) vs. 119 of 180 (66%), *p* = 0.004 and Grade 3: two of 25 (8%) vs. three of 180 (2%), *p* = 0.004. (Table 2).

**Table 2:** Comparison of patient and tumor characteristics in the actual clinical setting, where all patients had all undergone SLNB, according to postoperative sentinel lymph node status

Comparison of cohort characteristics	SLN Positive	SLN Negative	*p*-value
Number of patients	25	180	NA
Age, years (mean, range)	74 (66–88)	72 (65–89)	0.22^a^
Lymph node metastases, N (%)			
Micro-metastases	3 (12)	NA	NA
Macro-metastases	22 (88)	NA	NA
Types of surgery, N (%)			
Breast conservation	18 (72)	160 (89)	0.06^a^
Mastectomy	7 (28)	20 (11)	
Completion ALND, N (%)	12 (48)	NA	NA
With further nodal metastases	6 (50)	NA	NA

Comparison of final tumor biology	SLN Positive	SLN Negative	p-value
Tumor size, mean (range), mm	22 (10–45)	14 (1–47)	< 0.001^a^
Hormone receptor status, N (%)			
Positive	25 (100)	180 (100)	0.60^b^
Negative	0 (0)	0 (0)	
Ki-67 (cut-off ≤ 20%), N (%)			
Low	19 (76)	150 (83)	0.26^b^
High	6 (24)	30 (17)	
Nuclear Grade, N (%)			
1	1 (4)	58 (32)	0.004^b^
2	22 (88)	119 (66)	
3	2 (8)	3 (2)	
Intrinsic subtypes, N (%)			
Luminal A	18 (72)	143 (79)	0.57^b^
Luminal B	5 (20)	30 (17)	
HER-2 positive Luminal	2 (8)	7 (4)	

^a^ Independent T-test,

^b^ Fisher exact test. *P* < 0.05 was regarded as statistically significant

Abbreviations: *ALND* axillary lymph node dissection, *ER* estrogen receptor, *HER-2* human epidermal growth factor receptor 2, *NA *not applicable, *PR *progesterone receptor, *SLN *sentinel lymph node and *SLNB* sentinel lymph node biopsy

### Primary endpoints

#### Patients eligible for upfront SLNB omission only (Scenario A) and delayed-SLNB (Scenario B) 

**Fig. 2:**
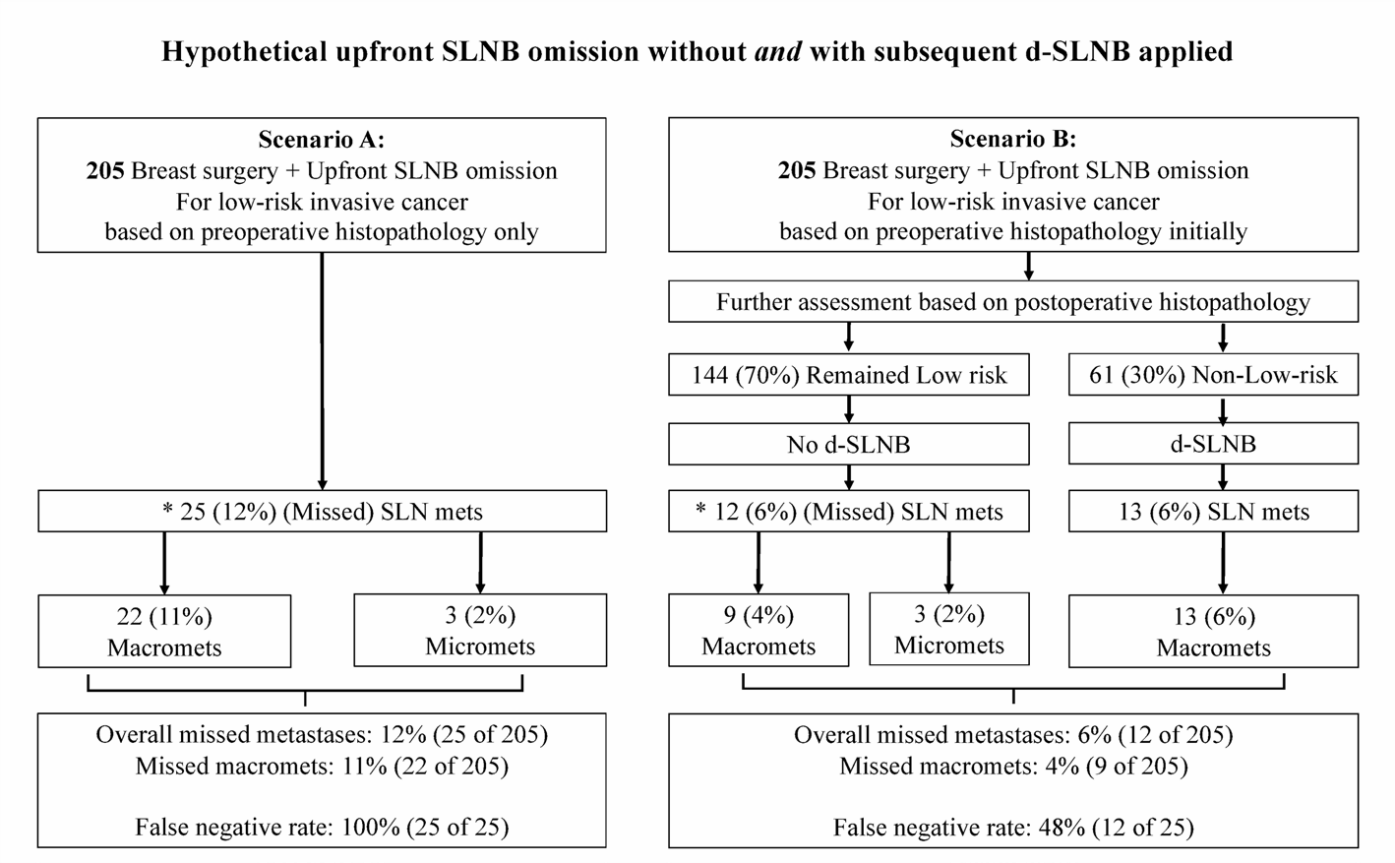
Analysis of outcome of axillary staging by hypothetically omitting SLNB in patients with low-risk invasive breast cancers based on preoperative histopathology only (Scenario A) and comparing to a further assessment based on postoperative histopathology to select patients for delayed SLNB (Scenario B). * Denoted SLN metastases that would have been missed if SLNB was omitted. Abbreviations: Macromets: macro-metastases, Micromets: micro-metastases, Mets: metastases, and d-SLNB: delayed sentinel lymph node biopsy

In the Actual clinical setting, of the 205 patients, 25 (12%) had SLN metastases, with three (2%) micro- and 22 (11%) macro-metastases, all would have been missed in Scenario A. However, in Scenario B, further assessment based on postoperative histopathology showed that 61 of 205 (30%) patients, were upstaged to non-low-risk breast cancers and therefore eligible for d-SLNB. This would lead to 13 of the 25 (52%) or (13 of all 205 (6%) patients with SLN metastases would have been detected. Whereas, amongst those 144 of 205 (70%) patients who remained low-risk on final histopathology in Scenario B, 12 (6%) had SLN metastases, of which nine (4%) with macro- and 3 (2%) with micro-metastases, would have been undetected. Therefore, upfront SLNB omission only (Scenario A) would have resulted in a proportion of 12% with missed SLN metastases compared to 6% if d-SLNB was added (Scenario B) (*p* < 0.001) (Fig. 2).

#### Changes in adjuvant treatments with upfront SLNB omission only (Scenario A) and with d-SLNB (Scenario B)

As per the low-risk cancer criteria, 180 patients expectedly did not have SLN metastases. Twenty-five patients had unexpected SLN metastases, three with micro-metastases only were regarded as SLN negative. Therefore, 22 of 25 (88%) patients with SLN macro-metastases were included in the analyses. The amount of given adjuvant treatments based on Actual clinical setting compared to Scenario A and B, respectively were as follow: completion ALND: 12 (55%) vs. 0 (0%) vs. 7 (32%), *p* < 0.001, endocrine therapy: 22 (100%) vs. 22 (100%) vs. 22 (100%), *p* = 0.99, bisphosphonates therapy: 18 (82%) vs. 0 (0%) vs. 10 (46%), *p* < 0.001, RT to breast or chest wall: 20 (91%) vs. 14 (64%) vs. 17 (77%), *p* = 0.10, axillary RT: 18 (82%) vs. 0 (0%) vs. 11 (50%), *p* < 0.001, and chemotherapy: 6 (27) vs. 0 (0%) vs. 6 (27%), *p* = 0.03. On average, 16 of 22 (73%) patients received adjuvant therapy in the Actual clinical setting compared to 6 of 22 (27%) if upfront SLNB omission only (Scenario A) and 12 of 22 (55%) if d-SLNB was applied (Scenario B) (Table 3). All differences in the amount of adjuvant treatments given between the Actual clinical setting and the hypothetical simulated Scenarios A and B were statistically significantly different except those of endocrine therapy and adjuvant breast or chest wall RT.

**Table 3:** Comparison of adjuvant treatments given to patients with macro-metastases based on the actual clinical setting where SLNB was actually performed, with two hypothetical scenarios: (A) upfront SLNB omission only and (B) upfront SLNB omission with d-SLNB

Types of adjuvant therapy	Actual Setting	Scenario A Hypothetical Simulation	Scenario B Hypothetical Simulation	
	Upfront SLNB Performed *N* = 22 (%)	Upfront SLNB* Omitted *N* = 22 (%)	Upfront SLNB* omitted with d-SLNB *N* = 22 (%)	*p*-value
Completion ALND	12 (55)	0 (0)	7 (32)	< 0.001
Endocrine therapy	22 (100)	22 (100)	22 (100)	0.999
Bisphosphonates therapy	18 (82)	0 (0)	10 (46)	< 0.001
Breast/Chest wall Radiotherapy	20 (91)	14 (64)	17 (77)	0.10
Axillary Radiotherapy	18 (82)	0 (0)	11 (50)	< 0.001
Chemotherapy	6 (27)	0 (0)	6 (27)	0.03
Average patients with macro-metastases who were given adjuvant therapy	16 (73)	6 (27)	12 (55)	< 0.001

Patients with lymph node macro-metastases were included in the analysis. * If SLNB omitted, SLN was considered free from metastases and all subsequent hypothetical treatments prescribed were based on Swedish National Guidelines [11]. § Chi square and Fisher’s Exact tests were used to compare proportions of adjuvant treatments. P-value of less than 0.05 was considered statistically significant. Abbreviations: ALND: axillary lymph node dissection, SLN: sentinel lymph node, SLNB: sentinel lymph node biopsy and d-SLNB: delayed sentinel lymph node biopsy

### Secondary endpoint

#### Costs of surgery

During the period of 2019 to 2021, the unit cost for BCS and mastectomy including SLNB was 5,500 EUR per patient. The unit cost for SLNB as part of the primary surgery was 500 EUR while a separate SLNB alone was 3,500 EUR, both included costs of SPIO injections. Based on 100 patients with low-risk invasive cancers who received treatment in the Actual clinical setting, the surgical cost was 550,000 EUR. If upfront SLNB omission only was instead adopted (Scenario A), the upfront saving would have been 50,000 EUR (100 SLNB avoided at unit cost of 500 EUR). However, if a d-SLNB implemented (Scenario B), approximately 30 of 100 patients (61 of 205, 30%, Fig. 2), would undergo standalone d-SLNB to stage the axilla, at a surgical cost of 105,000 EUR (30 d-SLNB at unit cost 3,500 EUR). Therefore, upfront SLNB omission with d-SLNB (Scenario B) would be associated with a total additional cost of 55,000 EUR (105,000 *minus* 50,000 EUR). However, with this additional cost, 70 (70%) patients would be spared the morbidities associated with axillary staging by using a selective d-SLNB approach at an extra unit cost of 785 EUR (55,000 EUR *divided* by 70).

## Discussion

The current study showed approximately 30% of patients, aged over 65 who underwent surgery for invasive breast cancers had low-risk invasive cancers that were clinically node negative and luminal A-like subtype. With retrospective simulation, where upfront SLNB was hypothetically omitted, 12% of SLN metastases would have been missed. However, if a d-SLNB was then selectively performed in patients with non-low-risk tumors based final postoperative histopathology, the proportion of missed SLN metastases would have been reduced by half (6%). Delayed-SLNB also led to statistically significantly more adjuvant treatments compared to upfront SLNB omission only. Despite the additional costs associated with d-SLNB, a majority of patients (70%) could avoid SLNB, thereby arm morbidities.

The incidence of low-risk luminal A subtype invasive cancers reported in the current study was comparable to those reported in the literature between 32 and 39% in the age group of 62 to 93 years [16]. The safety of omitting SLNB in this low-risk group has been reported in larger studies. A prospective observation cohort study by Ingvar et al. of low-risk patients in Sweden who were operated but did not undergo axillary staging between 1997 and 2002, showed a 15-years breast cancer specific survival at 93.7% with only 3% having axillary recurrence [17]. The recent SOUND trial showed the incidence of SLN metastases at 13.7%, which was similar to that of the current study, but omitting axillary surgery (including SLNB) was non-inferior to performing SLNB in terms of 5-year distant disease-free survival for patients with small breast cancers (up to 2 cm) and cN0 at diagnosis [5]. Adjuvant chemotherapy in the SOUND study was prescribed to similar proportions of patients who had a SLNB and those who did not, 20.1% vs. 17.5%, *p* = 0.22, respectively. This suggested that the axillary status probably did not affect the decision on adjuvant systemic treatment within the study cohort. However, in comparison, the proportions of patients who received chemotherapy in the current study was higher (27%) whereas if SLNB was omitted, less adjuvant chemotherapy would have been given. This suggested that d-SLNB could be a useful concept to be applied in these low-risk cancer patients as it was associated more adjuvant treatments compared to that of SLNB omission only. Arguably, in a more current context, gene expression tests like 21-Gene Expression Assay in Breast Cancer (Oncotype^®^) and 50 gene classifiers (PAM50) (Prosigna^®^) would have played a significant role in guiding decisions on systemic therapy [18, 19]. According to the current Swedish National Guidelines, nodal status continues to influence decisions on systemic therapy though the decision is increasingly assisted by gene expression profiling as well [20]. Therefore, despite recent trials with non-inferiority outcome after SLNB omission, we propose that a selective d-SLNB approach should still be considered in these low-risk cancer patients due to the potential additional clinically useful information of nodal status to guide adjuvant treatments and avoid undertreatment. Although selective d-SLNB in this study could lead to higher surgical costs, a large proportion of the low-risk patients, up to 70%, would still be spared of axillary surgery. Therefore, decision to perform d-SLNB should be based on potential benefits to the patient and not solely on cost considerations.

Conventionally, d-SLNB is performed by perioperative injections of lymphatic tracers like radioisotope or blue dyes. Although d-SLNB is proposed as a concept in this simulated study, it is also important to note its association with higher FNR compared to that of upfront SLNB due to alterations in lymphatic anatomy after surgery [21]. A potential method to circumvent the problem of FNR is to pre-mark the SLNs in advance at the primary operation. This can be done by injecting lymphatic tracers that stay longer in the SLNs until the indication for d-SLNB is subsequently confirmed based on the final histopathology. Longer lasting lymphatic tracers like indocyanine green dye, carbon nanoparticles and SPIO have been implicated [22–25]. However, the d-SLNB approach with SPIO should be weighed against cost-effectiveness of using these more expensive and longer lasting lymphatic tracers, which would have to be pre-injected in all patients. In that case, based on this current study, only one third of patients will eventually utilize the pre-injected tracers for d-SLNB therefore, with a negative implication on cost effectiveness.

This simulated study was not aimed to further demonstrate the non-inferiority in survival outcome of omitting upfront SLNB as larger studies have recently demonstrated that already. However, it was conceivable that there the survival outcome in this study cohort was also similar since our inclusion criteria were similar to the SOUND study (any age and tumor size up to 2 cm) [5, 6]. In that case, the actual clinical benefits in adopting d-SLNB could be questionably minimal. Nevertheless, we argue that delayed axillary staging with the d-SLNB technique can still be used selectively to guide adjuvant treatments. In addition to demonstrating reduction of FNR, perhaps further studies focused on the “number needed to treat” with d-SLNB in order to prevent one case of recurrence instead of, may be more clinically relevant and informative.

There are weaknesses associated with the current study besides the fact that analyses were based on a hypothetical simulated setting. The small cohort size limited the statistical power and generalizability of the findings. The retrospective data could be prone to biases where data collection may be incomplete. For example, tumor multifocality, a marker of tumor aggressiveness [15], could have been used to enhance the accuracy of selecting patients for d-SLNB. The hospital costs related to surgical procedures were only a retrospective approximation and the costs for surgery and postoperative care varied annually. In addition, formal health economic analysis for d-SLNB compared to standard care was not performed, therefore it was not possible to state the cost benefit outcome in SLNB omission from the health payer and patient perspective.

## Conclusion

This retrospective explorative study showed upfront SLN omission was suitable in almost 30% of patients in this study cohort who had preoperatively determined low-risk invasive cancers. However, there would be associated 12% risk in missing SLN metastases. If upfront SLNB was routinely omitted, selective d-SLNB could provide additional clinically useful SLN information that influence decision making in adjuvant treatments in a subgroup of low-risk breast cancer patients. Although recent studies indicated no difference in overall survival or distant disease-free survival if SLNB was omitted, the risks and benefits of leaving behind SLN metastases should still be carefully considered.

## Data Availability

Data availability The datasets used generated during and/or analyzed during the current study are available from the corresponding author on reasonable request.
